# Methamphetamine Disturbs Gut Homeostasis and Reshapes Serum Metabolome, Inducing Neurotoxicity and Abnormal Behaviors in Mice

**DOI:** 10.3389/fmicb.2022.755189

**Published:** 2022-04-18

**Authors:** Kai-Kai Zhang, Li-Jian Chen, Jia-Hao Li, Jia-Li Liu, Li-Bin Wang, Ling-Ling Xu, Jian-Zheng Yang, Xiu-Wen Li, Xiao-Li Xie, Qi Wang

**Affiliations:** ^1^Department of Forensic Pathology, School of Forensic Medicine, Southern Medical University, Guangzhou, China; ^2^Department of Toxicology, School of Public Health, Southern Medical University, Guangzhou, China

**Keywords:** methamphetamine, gut microbiome, serum metabolome, colonic inflammation, neurotoxicity

## Abstract

As an illicit psychostimulant, repeated methamphetamine (MA) exposure results in addiction and causes severe neurotoxicity. Studies have revealed complex interactions among gut homeostasis, metabolism, and the central nervous system (CNS). To investigate the disturbance of gut homeostasis and metabolism in MA-induced neurotoxicity, 2 mg/kg MA or equal volume saline was intraperitoneally (i.p.) injected into C57BL/6 mice. Behavioral tests and western blotting were used to evaluate neurotoxicity. To determine alterations of colonic dysbiosis, 16s rRNA gene sequencing was performed to analyze the status of gut microbiota, while RNA-sequencing (RNA-seq) and Western Blot analysis were performed to detect colonic damage. Serum metabolome was profiled by LC–MS analysis. We found that MA induced locomotor sensitization, depression-, and anxiety-like behaviors in mice, along with dysfunction of the dopaminergic system and stimulation of autophagy as well as apoptosis in the striatum. Notably, MA significantly decreased microbial diversity and altered the component of microbiota. Moreover, findings from RNA-seq implied stimulation of the inflammation-related pathway after MA treatment. Western blotting confirmed that MA mediated colonic inflammation by activating the TLR4-MyD88-NF-κB pathway and impaired colonic barrier. In addition, serum metabolome was reshaped after MA treatment. Specifically, bacteroides-derived sphingolipids and serotonin were obviously altered, which were closely correlated with locomotor sensitization, depression-, and anxiety-like behaviors. These findings suggest that MA disrupts gut homeostasis by altering its microbiome and arousing inflammation, and reshapes serum metabolome, which provide new insights into understanding the interactions between gut homeostasis and MA-induced neurotoxicity.

## Introduction

Methamphetamine (MA) is a highly addictive and illegal psychostimulant with serious health effects (Courtney and Ray, 2014; Centazzo et al., 2019). Based on a recent survey, there are about 24 million MA addicts worldwide, with over 57% of them being in South East Asia (Chomchai and Chomchai, 2015). Accumulating clinical and animal researches have shown that MA abuse can cause multiple abnormal behaviors, such as locomotor sensitization, depression, and anxiety (Courtney and Ray, 2014; Liu et al., 2019). Along with these behavioral phenotypes, neurotoxic effects of MA, including neuronal inflammation, apoptosis and autophagy, dopaminergic toxicity, and so on (Park et al., 2017; Shaerzadeh et al., 2018; Tan et al., 2020), have also been widely reported. Relatively, dopaminergic dysfunction, neural apoptosis and autophagy were reported in multiple locomotor sensitization and depression/anxiety animal models, while pharmacological inhibition of these neurotoxicity emerged the effective protection against these abnormal behaviors (Lin et al., 2016; Song et al., 2017; Banagozar et al., 2019; Meng et al., 2020). These findings imply that MA-induced locomotor sensitization, depression, and anxiety occur in tandem with dopaminergic dysfunction, neural apoptosis, and autophagy.

In the past decade, crucial roles of symbiotic microbiota in maintaining host homeostasis have been increasingly recognized, including immunity, metabolism, and the central nervous system (CNS; Chevalier et al., 2020). Alterations in microbiota have been profiled in depressed patients (Jiang et al., 2015), and this behavioral phenotype in patients can be transferred to mice through fecal microbiota transplant (Yang et al., 2020a). Moreover, probiotics supplementation has been shown to reduce depressive and anxiety symptoms in mice (Herman, 2019). Bacterial metabolites form the interactive link between microbiota and brain, and modulate depression and anxiety (Bercik et al., 2011; Sampson et al., 2016). Locomotor sensitization has also been shown to be closely associated with gut microbiota. In rodent models, quinpirole-induced locomotor sensitization was accompanied by microbial dysbiosis (Jung et al., 2018). Furthermore, antibiotic-mediated microbial reduction showed an enhanced sensitivity to cocaine-induced locomotor sensitization (Kiraly et al., 2016). In addition, emerging evidence reveals that intestinal immunity homeostasis impacts the CNS, including depression and anxiety behaviors. In inflammatory bowel disease (IBD), there is a heightened co-occurrence of depression and anxiety in patients, along with dysbiosis of microbiota (Abautret-Daly et al., 2018). Probiotic supplementation alleviates intestinal inflammation in IBD, thereby improving depression and anxiety (Emge et al., 2016). These findings have also been reported in high-fat-diet-induced depression- and anxiety-like behaviors. Microbial manipulation improved high-fat-diet-induced microbial dysbiosis and intestinal inflammation, which further alleviated behavioral impairments (Hassan et al., 2019; Zhao et al., 2019). These findings imply that there are complex and close relationships among microbiota, intestinal immunity, metabolism, and host behaviors.

Our previous study, which involved an escalating dose-multiple binge model, revealed that MA disturbs gut microbiota and metabolome, and shows potential regulatory effects on neurotoxicity (Chen et al., 2021). In a rat conditional place preference (CPP) model, MA was shown to restructure gut microbiota by lowering the abundance of the genus Phascolarctobacterium and elevating the abundance of the family Ruminococcaceae, and alter short-chain fatty acids in fecal samples (Ning et al., 2017). Moreover, microbial dysbiosis is involved in behavioral responses to MA abuse. Compared to rats with low CPP scores, rats with high scores showed different microbiota component, and pretreatment with antibiotics led to stronger CPP scores (Yang et al., 2020b). In addition, acute MA withdrawal disturbs microbiota composition and mediates depression-like behaviors (Forouzan et al., 2021). These findings imply the existence of microbiota-brain axis in MA-induced neurotoxicity.

Therefore, it is necessary to investigate the impacts of MA on gut homeostasis and neurotoxicity, and profile the potential metabolic pathways involved. In this study, biological measures and behavioral tests were performed to detect MA-induced neurotoxicity. To evaluate alterations in gut homeostasis, 16s rRNA sequencing analysis was done to analyze the status of gut microbiota, while RNA-seq and western blotting analyses were, respectively, performed to detect colonic damages. Finally, LC/MS analysis was conducted to profile serum metabolites.

## Materials and Methods

### Chemicals and Animals

Methamphetamine (purity > 99%) was obtained from the National Institute of Pharmaceutical and Biological Products Control (Beijing, China) and dissolved in 0.9% physiological saline to a concentration of 0.2 mg/ml.

Wild-type C57BL/6 male mice (8–10 weeks old) were purchased from the Experimental Animal Center of Southern Medical University and maintained in a standard animal room. Animal experiments were performed according to the guidelines provided by the National Institutes of Health Guide for the Care and Use of Laboratory Animals (Ethical Committee Approval Code: L2018123).

### Treatments

**Figure 1:**
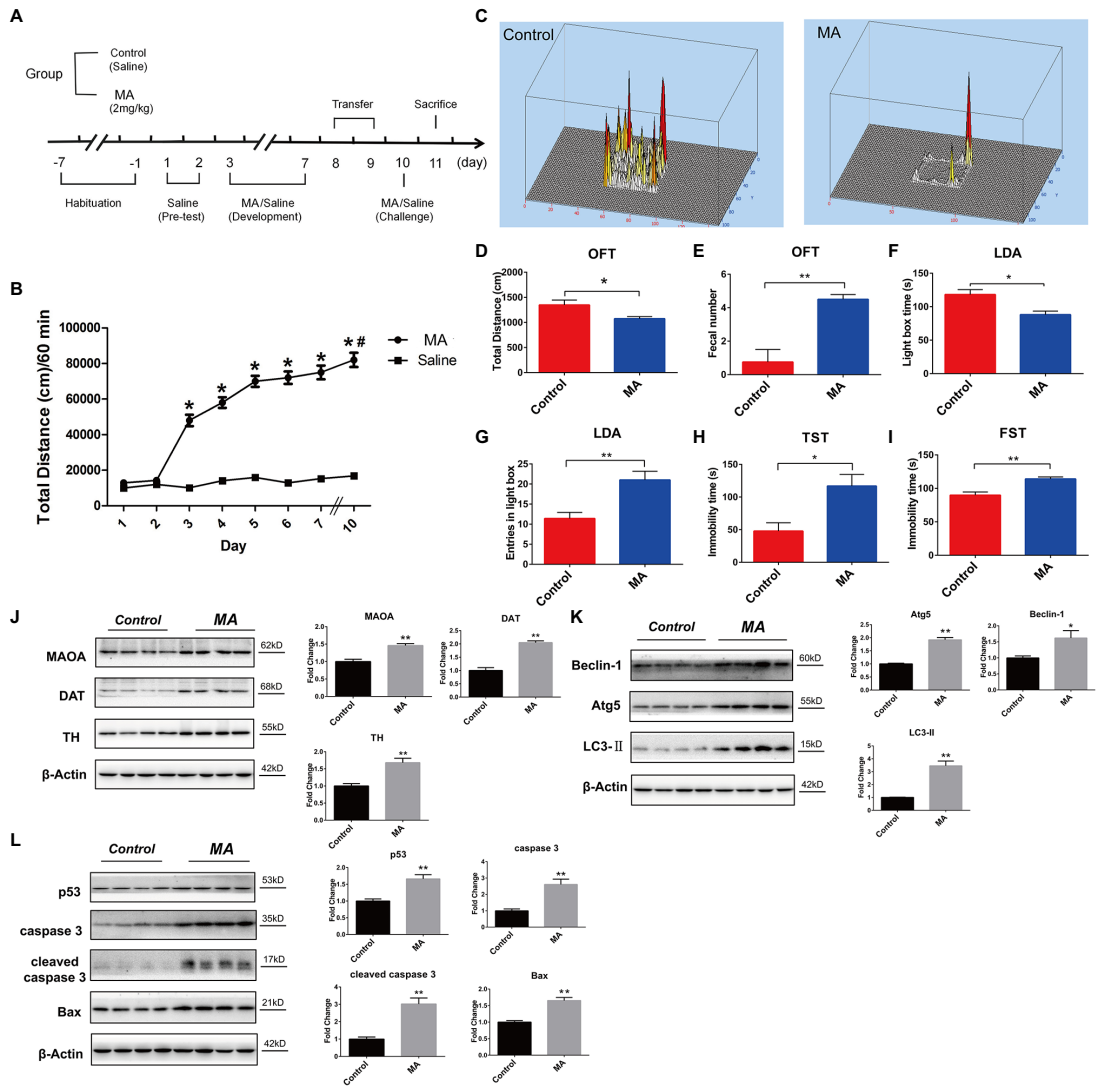
Methamphetamine (MA)-induced abnormal behaviors and striatal neurotoxicity in mice. **(A)** MA treatment schedule. **(B)** MA obviously elevated the locomotor activities, relative to the control group, ^*^p < 0.05; in MA group, 2 days’ interval further enhanced the locomotor activities in comparison with day 3, ^#^*p* < 0.05. Behavioral test. Open field test (OFT; **C–E**). MA obviously decreased total distance and increased fecal number when compared to the control group. Light–dark activity (LDA) test **(F,G)**. Mice spent less time in the light box and increased the entries in light box after MA treatment. Forced swimming test (FST; **H**) and Tail suspension test (TST; **I**). MA significantly increased immobility time of mice in the two tests when compared to the control group. Western blotting analysis. The expression levels of striatal dopamine transporter (DAT), tyrosine hydroxylase (TH), and monoamine oxidase A (MAOA; **J**), Beclin1, ATG 5 and LC3-II **(K)**; p53, caspase 3, cleaved caspase 3, and Bax **(L)** were higher in MA group than the control group. Data was expressed as the mean ± SEM after performing ANOVA, 0.01 < ^*^p < 0.05; 0.001 < ^**^p ≤ 0.01.

Mice locomotor sensitization model was established as previously described (Figure 1A; Liu et al., 2019). Briefly, 2 days before schedule (day 1–2, pre-test), all mice were pretreated by saline injection to test baseline locomotor activity. Then, mice were randomly allocated into the control or MA groups. Mice were continuously administered with intraperitoneal (i.p.) saline/MA (2 mg/kg, 24-h intervals) injections for 5 days (day 3–7, the development phase). After the transfer phase (day 8–9, free-injection interval), mice were administered with the same dose of saline/MA used in the development phase on day 10 (the expression phase). All injections were administered at the same time of day.

Subsequently, after collecting fecal samples, mice were anesthetized with 0.3% pentobarbital sodium (50 mg/kg i.p) after which blood samples were immediately collected. Then, mice were killed with an overdose of pentobarbital (60 mg/kg i.p; Xu et al., 2018). Colons and striatums were collected and frozen in liquid nitrogen at −80°C. Blood samples were stored for 30 min at room temperature and centrifuged (3,000 r/min, 10 min) to separate serum, which was then frozen for further analysis.

### Behavioral Tests

#### Locomotor Sensitization Test

Before being injected, mice were acclimatized by being placed in white floor and black wall (43 cm × 43 cm × 43 cm) open-field chambers for 30 min to adapt to the environment. After that, mice received i.p. injections. Smart Video tracking system (version 3.0; PanLab Technology for Bioresearch, Barcelona, Spain) was used to record and analyze mice locomotor activity for 60 min.

#### Open Field Test

Around 24 h after the last injections, open-field chambers were used to measure mice locomotor activities. Mice were allowed to freely explore the open area for 5 min. The number of fecal and total distance were recorded by Smart Video. Before each test, the device was thoroughly cleaned using 75% ethanol.

#### Light–Dark Activity Test

The device was composed of two equal size compartments (light compartment with white wall, dark one with black, 23.5 cm × 14 cm × 40 cm) and connected with an access (7 cm × 7 cm). Mice were originally placed in the dark compartment and allowed to freely explore the two boxes for 5 min. Entries and time spend in the light compartment were recorded by Smart Video. Compartments were thoroughly cleaned before each test.

#### Tail Suspension Test

Mice were, respectively, suspended 20 cm above the floor using a tape (1 cm distance from the tail tip) for 6 min. And mice activities were recorded by Smart Video during the last 4 min. Immobility time was considered as the index of depression-like behavior (hanging without body movement).

#### Forced Swimming Test

Mice were individually placed into a Plexiglas cylinder (25 cm high, 10 cm in diameter) that was filled with 10 cm water (temperature, 25°C). Each mouse was left in the cylinder for 6 min. Similarly, only the last 4 min were recorded to further analyze immobility time (floating without struggling and the necessary movements to keep its head above the water).

### 16S rRNA Gene Sequencing

Microbial DNA was extracted from fecal samples using the E.Z.N.A.® soil DNA Kit (Omega Bio-tek, Norcross, GA, United States) according to the manufacturer’s instructions. DNA concentrations and purity were determined using the NanoDrop 2000 UV–Vis spectrophotometer (Thermo Scientific, Wilmington, United States). Subsequently, we performed PCR using primers: 338F: 5′-ACTCCTACGGGAGGCAGCAG-3′ and 806R: 5′-GGACTACHVGGGTWTCTAAT-3′, which were used to amplify the V3–V4 regions of the bacterial 16S rRNA gene in an ABI GeneAmp® 9700 PCR thermocycler (ABI, CA, United States). Similarity cutoff of 97% was regarded as the screening index of operational taxonomic units (OTUs). Clustering was done using UPARSE (version 7.1; Edgar, 2013).^1^ After identification and removal of mismatched sequences, the taxonomy of each OTU representative sequence was analyzed using an RDP Classifier^2^ against the 16S rRNA database using a confidence threshold of 0.7 (Wang et al., 2007).

### Determination of MA Concentration

The concentration of MA in serum and fecal samples from MA group mice were determined using LC–MS/MS method. In brief, 1 ml serum or fecal suspension was intensively mixed with 2 ml 0.01 mol/L Na2B4O2 and 3 ml diethyl ether. Afterward, mixtures were centrifuged (13,000 r/min, 10 min) to separate the supernatant and then, volatilized to dryness at 45°C. Samples were resolubilized with 200 μl of 70% acetonitrile solution and filtered using a 0.22 μm filter membrane. An LC-20AD Prominence UFLC system (Shimadzu, Japan) coupled with a Z-Spray electrospray interface was used to perform the chromatographic separation. Injections were made onto an Agilent Zorbax SB C18 chromatographic column (150 mm × 2.1 mm, 3.5 μm) in this study (CA, United States). The detailed procedures and parameters were set as described previously (Milesi-Hallé et al., 2015; Zhang et al., 2019).

### RNA-Isolation and Sequencing, Read Mappings, Differential Expression Analysis, and Functional Gene Annotations of the Colon

Briefly, the TRIzol reagent was used to isolate total RNA according to the manufacture’s instructions. NanoDrop2000 (Thermo Fisher scientific, America) was performed to guarantee the high quality of RNA samples (OD260/280 = 1.8–2.2, OD260/280 ≥ 2.0, RIN ≥ 6.5, 28S/18S ≥ 1.0, >10 μg). The sequencing library was constructed using the RNA preparation kit (Illumina, San Diego, CA, United States) and the purified cDNA was sequenced on the Illumina HiSeqTM 2500 platform (Sengupta et al., 2011).

Sequencing reads were trimmed and quality controlled by SeqPrep^3^ and Sickle.^4^ Filtered reads were mapped to the reference genome using the TopHat software (version2.0.0; Trapnell et al., 2009).^5^ To identify differentially expressed genes (DEGs) between the control and MA groups, Fragments per Kilobases per Million (FPKM) reads method was used to analyze gene expressions across samples (P-adjust < 0.05, fold-change ≥ 2 or ≤0.5).

Screened DEGs were annotated by Gene ontology (GO) analysis using online GO Resource,^6^ according to the functional classification of biological processes, cellular components, and molecular functions (Ashburner et al., 2000). For multi-dimensional annotation, DEGs were further mapped to the Kyoto Encyclopedia of Genes and Genomes (KEGG)^7^ based on the first category [metabolism (M), environmental information processing (EIP), cellular processes (CP), organismal systems (OS), and human diseases (HD); Kanehisa et al., 2017]. Then, protein–protein interaction (PPI) tools (Cytoscape_v3.6.1)^8^ were performed to reveal the interactions among DEGs (Aihaiti et al., 2021). Finally, pathway enrichment analyses were conducted on the DAVID Bioinformatics Resources^9^ to gather the detailed pathway messages (Tan et al., 2020).

### LC–MS/MS Analysis

To extract pure metabolites, the mixture of 100 μl serum sample and 400 μl methanol solution (80%) was subjected to a high-throughput tissue crusher Wonbio-96c (Shanghai Wanbo Biotechnology Co., LTD) after which the supernatants were obtained by centrifugation (13,000 *g*, 4°C, 15 min) for further LC–MS/MS analysis.

The ExionLCTMAD system (AB Sciex, United States) equipped with an ACQUITY UPLC BEH C18 column (100 mm × 2.1 mm i.d., 1.7 μm, Waters, Milford, United States) was used to separate the metabolites. A pooled quality control sample was used to reflect analytical stability. Mass spectrometric data were acquired using a Thermo UHPLC-Q Exactive Mass Spectrometer. Progenesis QI 2.3 (Nonlinear Dynamics, Waters, United States) was used to analyze the raw data based on UPLC-TOF/MS analyses. MS/MS fragments score > 30 was considered as confidently identified for metabolic confirmation. Based on the above-mentioned metabolic features, MS/MS information was matched to the human metabolome database (HMDB)^10^ and Metlin database (Dunn et al., 2011).^11^

### Western Blotting

A western blot assay was performed to investigate neurotoxicity and colonic toxicity associated with MA. The RIPA lysis method was used to isolate total proteins of striatum and colonic mucosa as previously described (Zhang et al., 2021). The primary antibodies used were: anti-monoamine oxidase A (MAOA; 1:1,000, 4A Biotech, Cat# 4ab091383), anti-dopamine transporter (DAT; 1:1,000, Bioss, Cat# bs-1714R), anti-tyrosine hydroxylase (TH; 1:1,000, Proteintech, Cat# 25859-1-AP), anti-caspase 3 (1:1,000, ABclonal, Cat# A2156), anti-cleaved caspase 3 (1:1,000, CST, Cat# 9661), anti-p53 (1:1,000; 4A Biotech, Cat# 4ab087781), anti-Bax (1:1,000, CST, Cat# 2772), anti-Beclin 1 (1:1,000, CST, Cat# 3738), anti-LC3-II (1:1,000; Proteintech, Cat# 18725-1-AP), anti-autophagy-related 5 (Atg5; 1:1,000; HuaAn Biotechnology, Cat# ET1611-38), anti-Claudin-5 (1:1,000, Abclonal, Cat# A10207), anti-Occludin (1:1,000, Abclonal, Cat# A2601), anti-TLR4 (1:1,000, SANTA, Cat# sc-293072), anti-MyD88 (1:1,000, CST, Cat# 4283S), anti-NF-κB (1:1,000, Proteintech, Cat# 10745-1-AP), anti-NLRP3 (1:1,000, Abcam, Cat# ab263899), anti-IL-6 (1:1,000, 4A Biotech, Cat# 4ab044340), anti-IL-18 (1:1,000, Proteintech, Cat# 60070-1-lg), anti-TNF-α (1:1,000, 4A Biotech, Cat# 17590-1-AP), anti-caspase 1 (1:1,000, Proteintech, Cat# 22915-1-AP), and anti-β-actin (1:1,000; 4A Biotech, Cat# 4ab071291). Secondary antibodies of either HRP-labeled goat anti-mouse IgG (H + L; 1:5,000, Cat# IH-0031) or HRP-labeled goat anti-rabbit IgG (H + L; 1:5,000, Cat# IA-0072) were purchased from Beijing Dingguo Changsheng Biotechnology.

### Statistical Analysis

For statistical analysis, one-way repeated measures ANOVA was performed for locomotor sensitization test, while other behavior tests and Western blot were analyzed using the student’s *t*-test method (Graphpad Prism, version 6.0 or SPSS version 17). All experiments were performed in triplicate at the minimum. 16S rRNA sequencing data were analyzed using the following methods: Unweighted Pair-group Method with Arithmetic Mean (UPGMA) for Principal co-ordinate analysis (PCoA) and Hierarchical clustering tree; Wilcoxon rank-sum test referring to Mann–Whitney U tests (CI was set at 95%) for screening of differential microbiota; non-parametric factorial Kruskal–Wallis (KW) sum-rank test for LEfSe cladogram. For RNA Sequence analysis and LC–MS/MS analysis, the eigenvalue of covariance matrix method was utilized for principal component analysis (PCA); Spearman analysis was utilized for correlation analysis among samples. In the orthogonal least partial squares discriminant analysis (OPLS-DA) model, the Student’s *t*-test was used to evaluate significant differences between the control and MA groups. The variable importance plot (VIP) was also analyzed based on OPLS-DA method. In addition, Spearman analysis was applied to correlation analysis between differential microbiota and metabolites. Pathway enrichment analyses were carried out through Topologic method (Relative-betweenness Centrality) on the DAVID Bioinformatics Resources, based on the KEGG. All results are presented as the mean ± SEM. Value of *p* < 0.05 indicated statistical significance.

## Results

### MA-Induced Neurotoxicity in Mice

#### MA-Induced Locomotor Sensitization and Depression/Anxiety-Like Behaviors in Mice

Compared to saline treatment, MA injections from day 3 significantly increased the total distance traveled (*p* < 0.01), and this was sustained throughout the development phase (Figure 1B). After 2-day withdrawal (day 8–9), re-exposure of MA on day 10 enhanced mice hyper-activity when compared to the first exposure on day 3 (*p* < 0.05), implying that 2 mg/kg MA induced locomotor sensitization.

In the open field test (OFT), compared to the control group, total distance was significantly reduced after MA treatment (Figures 1C,D), mirroring the lower locomotor activity. In both tail suspension test (TST) and forced swimming test (FST), MA significantly elevated immobility time of mice when compared to the control group (Figures 1H,I). These results indicate higher depression levels in mice after MA treatment.

Methamphetamine treatment significantly elevated fecal number in relative to the control group during OFT (Figure 1E). Similarly, in the light–dark activity (LDA) test, mice spent less time in the light compartment after MA administration (Figure 1F), suggesting an increased anxiety level in mice of the MA group. Interestingly, MA obviously increased entries in the light compartment in comparison with the control group (Figure 1G), which meant the less time of each entry in the MA group and mirrored the higher anxiety of mice.

#### MA-Induced Dopaminergic Damage, Apoptosis- and Autophagy-Related Neurotoxicity in Mice Striatums

Given the close association between striatal dopamine (DA) and MA-induced locomotor sensitization (Fukushima et al., 2007), we examined dopaminergic system functions. MA significantly upregulated the expression levels of DAT, TH, and MAOA, indicating a disturbance in DA metabolism (Figure 1J).

Methamphetamine significantly elevated the expression levels of Beclin 1, ATG 5, and LC3-II in striatums, which were associated with autophagy (Figure 1K). Furthermore, levels of apoptosis-related proteins, including p53, Bax, caspase 3, and cleaved caspase 3, were higher in the MA group than in the control group (Figure 1L).

### MA Disturbed Gut Microbiome

#### Effects of MA on Microbial Richness and Diversity

**Figure 2:**
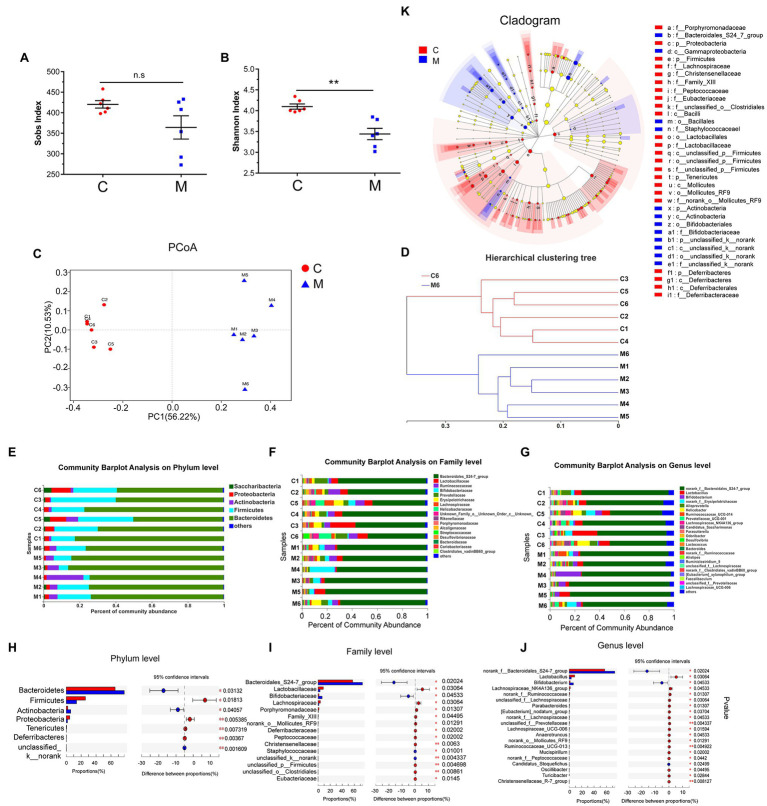
Methamphetamine induced the disturbance of gut microbiome in mice. Sobs **(A)** and Shannon indices **(B)** reflect the alterations of microbial diversity and richness between the control and MA groups. Microbial structures of MA (blue) and control samples (red) were assessed by Principal co-ordinate analysis (PCoA; **C**) and Hierarchical clustering tree **(D)** using Unweighted Pair-group Method with Arithmetic Mean (UPGMA). Community Barplot analysis showed the microbial composition and relative abundance of all samples on Phylum, Family, and Genus level **(E–G)**, while the differential microbiota at each taxon between two groups was screened using Wilcoxon rank-sum test referring to Mann–Whitney U tests (CI was set at 95%; **H–J**). LEfSe Cladogram further showed the differential microbiota at different taxon **(K)**, 0.01 < ^*^p < 0.05; 0.001 < ^**^p ≤ 0.01.

Based on the OTUs analysis of 16S rRNA sequencing, Alpha-diversity was performed using the Student’s *t*-test to evaluate microbial richness and diversity. Regarding microbial richness, there were no significant changes in the Sobs index, Ace index, and Chao index (Figure 2A; Supplementary Figures S1A,B), suggesting that microbial richness was the same in both groups. Compared to the control group, MA significantly reduced microbial diversity, as indicated by a lower Shannon index (4.099 ± 0.153 vs. 3.440 ± 0.334; Figure 2B) and a higher Simpson index (0.0366 ± 0.0077 vs. 0.1030 ± 0.0461; Supplementary Figure S1C).

#### MA-Induced Alterations in Microbial Composition

Unweighted PCoA integrally revealed alterations in microbial community composition in Beta-diversity. Based on sample distance, MA samples were distinctly separated from control samples. Samples in the control group exhibited a higher similarity than those in the MA group (Figure 2C). Based on the distance matrix of Beta-diversity, hierarchical clustering was performed to assess interindividual similarity at the OTU level. Samples in the MA and control groups were clustered separately (Figure 2D), indicating that there was a similarity in the intra-group and differences in the between-group community composition.

To identify MA-induced microbiota alterations, taxonomic analysis was performed to reveal the structure of microbial community and the relative abundance of microbiota in all samples at the phylum, family, and genus levels (Figures 2E–G). Accordingly, the Wilcoxon rank-sum test referring to Mann–Whitney U tests was used to further identify the different microbiota between the MA and control groups, based on the relative microbiota abundance. At the phylum level, the abundance of seven main microbiota were altered in the MA group in comparison with the control group. This included the elevation of Bacteroidetes, Actinobacteria, and unclassified k_norank, and the lower Firmicutes, Proteobacteria, Tenericutes, and Deferribacteres (Figure 2H). Moreover, we screened the top 20 different microbiota at the family and genus levels for further analyses (Figures 2I,J). The cladogram revealed a number of different features through LEfSe analysis (Figure 2K). MA elevated the abundance of the probiotic Bifidobacteriaceae at the family and genus level; however, the abundance of the family and genus Lactobacillaceae was decreased following MA treatment. MA also reduced the abundance of Lachnospiraceae and elevated the abundance of the Bacteroidales_S24-7-group at the family level. The abundance of Bifidobacterium at the genus level was significantly higher after MA treatment. In contrast, the abundance of norank_f_Ruminococcaceae and Ruminococcaceae_UCG-013 (family Ruminococcaceae), as well as the Lachnospiraceae_NK4A136_group, Lachnospiraceae_UCG-006, norank_f_, and unclassified_f_ Lachnospiraceae (family Lachnospiraceae) were reduced following MA treatment. To exclude the adverse effects of MA in feces, we further detected the concentration of MA in feces and serum after MA treatment, through LC–MS/MS method. The results showed that MA was not detected in feces, but was detected in serum (MA: 0.898 ng/ml ± 0.12106 ng/ml, mean ± SEM, *N* = 5). These results further indicated that MA-induced disturbance of the microbiota was independent of MA in feces.

### MA Resulted in the Inflammation on Colon

#### RNA-Sequencing Analysis Revealed the Pro-inflammatory Effects of MA on Colons

Given the close association with microbiota, RNA-sequencing analysis was performed to detect colon impairment.

##### Similarity Analysis Among Samples

**Figure 3:**
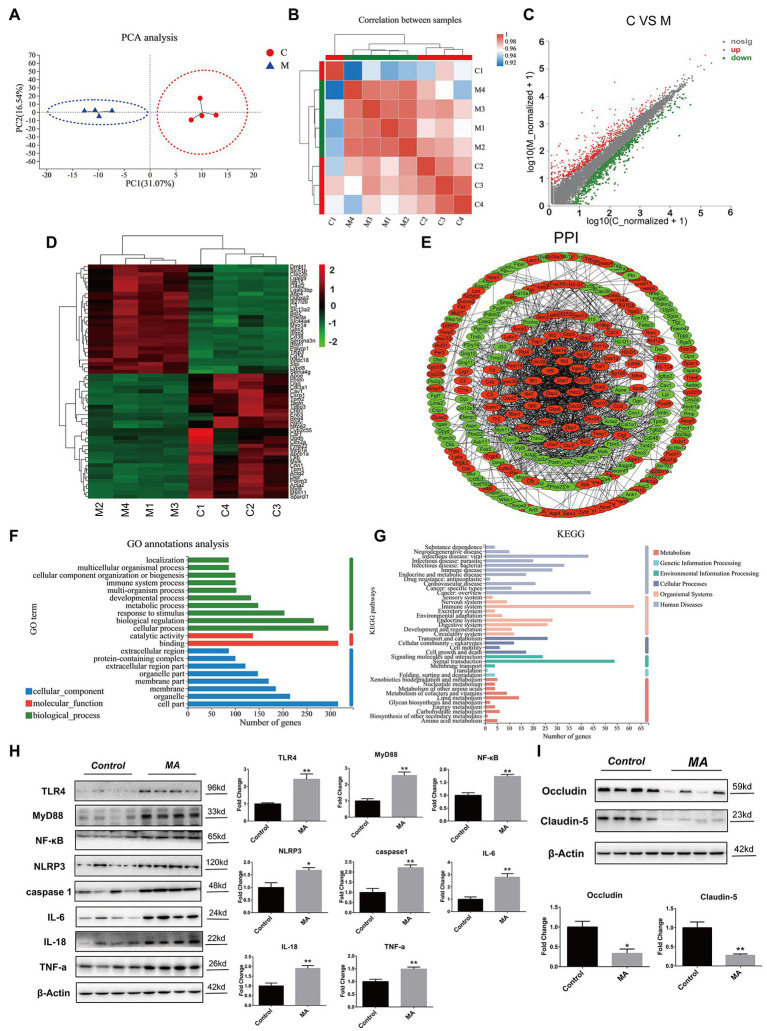
Methamphetamine stimulated inflammation and damaged barrier function in colon. Principal component analysis (PCA) analysis **(A)**. Samples in the control and MA groups were, respectively, clustered. Correlation analysis **(B)**. The tree represents correlations among samples, and the color represents the degree of correlation. Identification of differentially expressed genes (DEGs) using the Scatter plot method **(C)**. Compared to the control group, MA treatment resulted in 414 DEGs (fold change ≥ 2 or ≤0.5, value of *p* < 0.05). Increased DEGs are labeled with red plots, whereas suppressed DEGs are shown in the green plots. Hierarchical clustering of the top 60 DEGs **(D)**. The tree depicts log10 transformation of average fold changes. Green represents downregulated genes, whereas red denotes upregulated genes. Functional annotations of DEGs. Protein–protein interaction (PPI) analysis of DEGs **(E)**. MA-induced upregulated DEGs were depicted by red, while downregulated DEGs were displayed by green. Gene Ontology (GO) annotation analysis of DEGs **(F)**. DEGs were enriched in 10 biological processes (green), eight cellular components (blue), and two molecular functions (red). Kyoto Encyclopedia of Genes and Genomes (KEGG) pathway enrichment analysis of DEGs **(G)**. DEGs were enriched in 10 metabolisms (red), two Genetic information processing (GIP; light blue), three environmental information processes (green), four cellular processes (CP; dark gray), nine organismal systems (OS; pink), and 11 human diseases (HD; light gray). A western blotting analysis demonstrating colonic expressions of TLR4, MyD88, NF-κB, NLRP3, caspase 1, TNF-α, IL-6, IL-18, Occludin, and Claudin-5 proteins. In contrast with the control group, MA significantly upregulated the expression levels of TLR4, MyD88, NF-κB, NLRP3, caspase 1, TNF-α, IL-6, and IL-18 **(H)**. Of note, MA markedly inhibited Occludin and Claudin-5 protein levels (**I**; 0.01 < ^*^p < 0.05; 0.001 < ^**^p ≤ 0.01).

Through simplifying RNA-seq reads, the PCA can integrally mirror the similarity of sample components (Giuliani, 2017). The closer the samples, the more similar they are. In this study, samples in the control (red spots) and MA groups (blue triangles) were clustered, respectively (Figure 3A). In the correlation analysis, intra-group samples also exhibited higher correlations and were clustered, except sample C1 (Figure 3B), implying that principal components of the intra-group samples were similar, while the inter-group samples were different.

##### Screening of the DEGs

Compared to the control group, MA induced 414 DEGs, including 189 upregulated DEGs and 225 downregulated DEGs (Figure 3C). Among these DEGs, the top 60 DEGs (30 upregulated and 30 downregulated DEGs) were integrated through hierarchical cluster analysis (Figure 3D).

##### Functional Annotations of the DEGs

Protein–protein interaction analysis elucidated on the interactions among the DEGs. MA-induced DEGs, which have the direct or indirect relations, were linked, while unidentified or irrelevant DEGs were eliminated. Upregulated DEGs were marked by red, while downregulated ones were labeled with green (Figure 3E).

Based on functional classification, GO annotation classified these DEGs into three main groups, including 10 biological processes (e.g., cellular process, biological regulation and response to stimulus), eight cellular components (e.g., cell part, organelle, and membrane) and two molecular functions (e.g., binding and catalytic activity; Figure 3F).

In addition, KEGG enrichment analysis was performed for the identification of pathways. Around 414 DEGs were mainly enriched into 29 pathways (value of *p* < 0.05). Among these pathways, inflammation-related pathways were mostly enriched, such as Herpes simplex infection, Phagosome, Staphylococcus aureus infection, Focal adhesion, TNF signaling pathway, and IBD (Supplementary Table 1). Then, the DEGs were further annotated following the classification of metabolism, EIP, CP, OS, and HD. In human diseases, infectious diseases (viral, parasitic, and bacterial) and immune diseases were mostly enriched. Notably, substance dependence and neurodegenerative diseases were also enriched, which were associated with MA addiction as well as neurotoxicity. Correspondingly, in organismal systems, the immune system was the most influenced system (Figure 3G). These findings show the pro-inflammatory effects of MA on colons.

#### MA Promoted Colonic Inflammation by Stimulating the TLR4-MyD88-NF-κB Pathway, and Damaged Colonic Barrier Functions

The Gram-negative bacterial product-lipopolysaccharide (LPS) is well-known as the stimulation of Toll-like receptor 4 (TLR4; Płóciennikowska et al., 2015). Given the alterations of microbiota after MA treatment, we aimed at determining whether the TLR4 pathway was activated and further mediated colonic inflammation. Indeed, MA significantly elevated the expression of TLR4, MyD88, NF-κB, and NLRP3 in comparison with the control group. Inflammatory factors, including TNF-α, caspase 1, IL-18, and IL-6, were also found to be upregulated after MA treatment (Figure 3H). These findings confirm that MA induced colonic inflammation by stimulating the TLR4-MyD88-NF-κB pathway.

Given the close association between colonic inflammation and barrier dysfunctions (Shen et al., 2020), expression levels of tight junction proteins were also determined. The expression levels of Occludin and Claudin-5 in the MA group were lower than in the control group (Figure 3I), indicating a weakened intestinal barrier.

### MA Altered Serum Metabolome in Mice

#### Effects of MA on Serum Metabolic Composition

**Figure 4:**
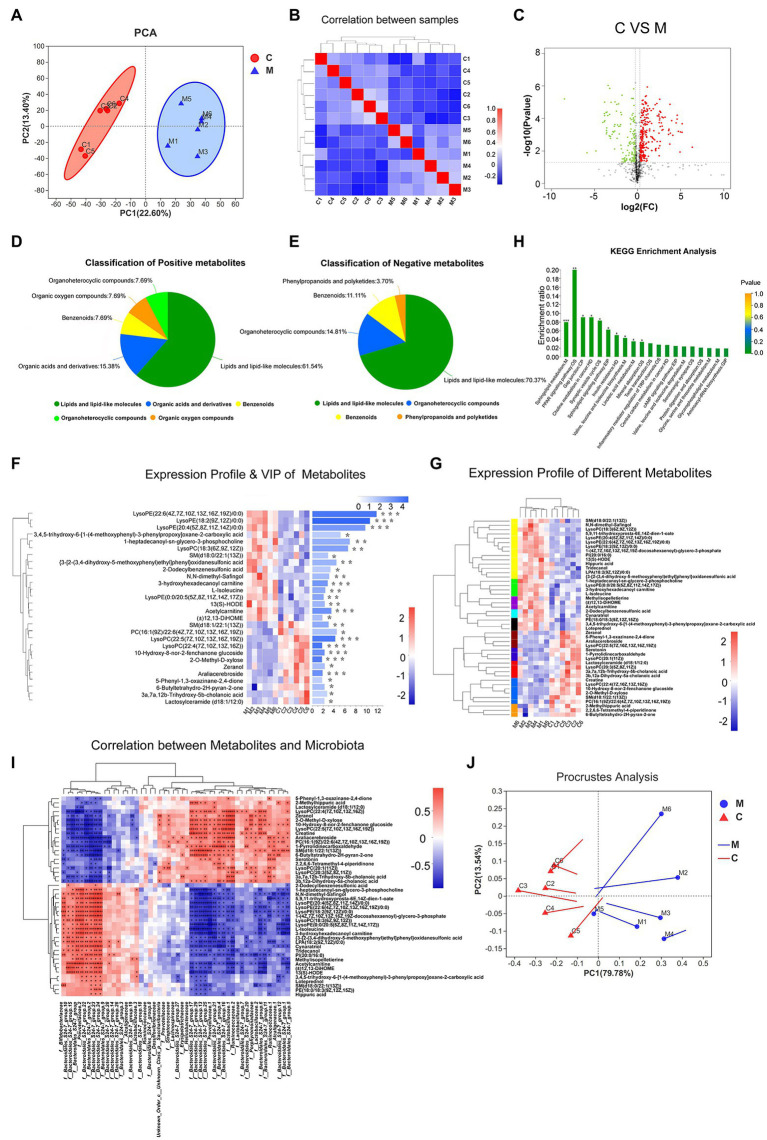
The metabolic profiles of various samples. PCA analysis **(A)** and correlation analysis **(B)** showed the structure and similarity of metabolic composition among samples (confidence level over 95%). Identification of differential metabolites using Volcano plot analysis **(C)**. Up-regulated metabolites are marked by red plots, while down-regulated are presented by green plots. Based on human metabolome database (HMDB) compound, Pie plots show the classification of positive and negative metabolites **(D,E)**. Expression profile and VIP of differential metabolites **(F,G)**. Lattice color of the left heatmap represents metabolite abundance and metabolites with similar expressing patterns were clustered by tree. The corresponding VIP values were shown in the right bar chart. Metabolic KEGG analysis **(H)**. Acronyms denote the classification of metabolic pathways. M, metabolism; OS, organismal systems; EIP, environmental information processing; CP, cellular processes; HD, human diseases; and GIP, genetic information processing. According to the relative abundance, correlation between differential microbiota and metabolites was performed by heatmap using Spearman correlation analysis **(I)**. Similarly, Procrustes analysis showed variations between microbiota and metabolites. Solid dots of line segment were on behalf of microbiota data and the other end denoted metabolic data (**J**; 0.01 < ^*^p < 0.05; 0.001 < ^**^p ≤ 0.01; and ^***^p ≤ 0.001).

We performed UPLC-TOF/MS analyses to determine the status of serum metabolome. PCA scores provided a valid measure to globally overview the composition of samples. MA samples were separated from the control group and grouped into clusters. All the samples were within the 95% confidence level (Figure 4A). Correlation analysis of samples revealed a high similarity in the intra-group and a significant difference in the inter-group (Figure 4B).

To filter MA-induced differential metabolites, the following criteria were used: VIP > 1, fold change > 1.2 or <0.83, value of *p* < 0.05 (Figure 4C). Subsequently, 425 differential metabolites between the MA and control groups were screened. These included 165 positively ionized metabolites and 260 negatively ionized metabolites. Among these metabolites, only 15 positively and 31 negatively ionized metabolites could be annotated. Based on the HMDB compounds database, 13 positively and 27 negatively ionized metabolites were successfully classified (Figures 4D,E). Among them, 27 metabolites belonged to Sphingolipids (4), Prenol lipids (3), Steroids and steroid derivatives (3), Fatty Acyls (3) and Glycerophospholipids of Lipids and lipid-like molecules (14); eight metabolites belonged to Piperidines (2), Carboxylic acids and derivatives (2), Lactones (1), Organooxygen compounds (1), Pyrrolidines (1), and Indoles and derivatives of Organic acids and derivatives (1); four metabolites belonged to Benzene and substituted derivatives of Benzenoids while one metabolite belonged to Macrolides and analogs of Phenylpropanoids and polyketides (Table 1).

**Table 1:** Differential metabolites.

Metabolite name	HMBD class	VIP	Flod change	RT (min)	lon (m/z)	Formula	*p*-value	pos/neg
*Lipids and lipid-like molecules*								
SM(d18:0/22:1(13Z))	Sphingolipids	3.08	1.25	13.72	831.64	C45H91N2O6P	<0.05	ESC −
Araliacerebroside		1.81	0.80	11.08	776.55	C40H77NO10	<0.05	ESC −
Lactosylceramide (d18:1/12:0)		2.06	0.35	9.44	850.55	C42H79NO13	<0.05	ESC −
SM(d18:1/22:1(13Z))		3.16	0.64	12.58	829.65	C45H89N2O6P	<0.05	ESC −
Cynaratriol	Prenol lipids	1.06	1.59	3.72	317.11	C15H22O5	<0.05	ESC −
5,9,11-trihydroxyprosta-6E,14Z-dien-1-oate		1.26	1.46	7.96	576.21	C30H37NO8	<0.05	ESC −
10-Hydroxy-8-nor-2-fenchanone glucoside		2.21	0.08	4.09	297.13	C15H24O7	<0.05	ESC −
Loteprednol	Steroids and steroid derivatives	1.13	1.76	2.56	436.19	C21H27ClO5	<0.05	ESC +
3a,7a,12b-Trihydroxy-5b-cholanoic acid		2.67	0.29	6.56	453.29	C24H40O5	<0.05	ESC −
3b,12a-Dihydroxy-5a-cholanoic acid		1.32	0.66	6.66	437.29	C24H40O4	<0.05	ESC −
3-hydroxyhexadecanoyl carnitine	Fatty acyls	2.74	1.48	6.90	398.33	C23H45NO5	<0.05	ESC +
Acetylcarnitine		3.34	1.51	0.80	204.12	C9H17NO4	<0.05	ESC +
Tridecanol		1.03	1.27	8.67	242.25	C13H28O	<0.05	ESC +
LysoPE[20:4(5Z,8Z,11Z,14Z)/0:0]	Glycerophospholipids	9.70	1.25	7.90	500.28	C25H44NO7P	<0.05	ESC −
PC{16:1[9Z]/22:6(4Z,7Z,10Z,13Z,16Z,19Z)}		4.08	0.78	10.38	848.55	C46H78NO8P	<0.05	ESC −
LysoPC[20:1(11Z)]		1.13	0.81	8.85	594.38	C28H56NO7P	<0.05	ESC −
LysoPC[22:4(7Z,10Z,13Z,16Z)]		1.76	0.72	8.33	616.36	C30H54NO7P	<0.05	ESC −
LysoPC[20:3(5Z,8Z,11Z)]		0.93	0.64	8.29	590.35	C28H52NO7P	<0.05	ESC −
LysoPC[22:5(7Z,10Z,13Z,16Z,19Z)]		4.24	0.60	8.12	614.35	C30H52NO7P	<0.05	ESC −
LysoPE[0:0/20:5(5Z,8Z,11Z,14Z,17Z)]		2.18	1.43	8.11	544.27	C25H42NO7P	<0.05	ESC −
LysoPE[18:2(9Z,12Z)/0:0]		10.34	1.41	7.97	476.28	C23H44NO7P	<0.05	ESC −
LysoPE[22:6(4Z,7Z,10Z,13Z,16Z,19Z)/0:0]		11.86	1.29	7.80	524.28	C27H44NO7P	<0.05	ESC −
1-(4Z,7Z,10Z,13Z,16Z,19Z-docosahexaenoyl)-glycero-3-phosphate		1.16	1.56	7.52	524.28	C25H39O7P	<0.05	ESC +
LPA[18:2(9Z,12Z)/0:0]		1.58	1.80	8.76	435.25	C21H39O7P	<0.05	ESC +
LysoPC[18:3(6Z,9Z,12Z)]		6.37	1.27	7.55	518.32	C26H48NO7P	<0.05	ESC+
PE[18:0/18:3(9Z,12Z,15Z)]		1.46	1.25	11.67	724.53	C41H76NO8P	<0.05	ESC +
PI(20:0/16:0)		1.10	1.97	7.78	903.53	C45H87O13P	<0.05	ESC −
*Organoheterocyclic compounds*								
6-Butyltetrahydro-2H-pyran-2-one	Lactones	2.29	0.64	4.76	201.11	C9H16O2	<0.05	ESC −
2-O-Methyl-D-xylose	Organooxygen compounds	1.75	0.07	3.73	165.08	C6H12O5	<0.05	ESC +
1-Pyrrolidinecarboxaldehyde	Pyrrolidines	1.29	0.25	5.44	243.13	C5H9NO	<0.05	ESC −
Serotonin	Indoles and derivatives	1.07	0.81	1.47	177.10	C10H12N2O	<0.05	ESC +
2,2,6,6-Tetramethyl-4-piperidinone	Piperidines	1.03	0.61	4.69	200.13	C9H17NO	<0.05	ESC −
Methylisopelletierine		0.91	1.62	5.19	200.13	C9H17NO	<0.05	ESC −
Creatine	Carboxylic acids and derivatives	1.38	0.68	0.69	132.07	C4H9N3O2	<0.05	ESC +
L-Isoleucine		2.31	1.25	1.22	132.10	C6H13NO2	<0.05	ESC +
Benzenoids								
Hippuric acid	Benzene and substituted derivatives	1.55	1.55	2.77	178.05	C9H9NO3	<0.05	ESC −
2-Dodecylbenzenesulfonic acid		3.40	1.32	9.04	325.18	C18H30O3S	<0.05	ESC −
5-Phenyl-1,3-oxazinane-2,4-dione		1.68	0.40	3.94	226.03	C10H9NO3	<0.05	ESC −
2-Methylhippuric acid		1.47	0.70	2.92	194.08	C10H11NO3	<0.05	ESC +
*Phenylpropanoids and polyketides*								
Zeranol	Macrolides and analogs	2.31	0.51	6.62	643.35	C18H26O5	<0.05	ESC −

Thirteen positively and 27 negatively ionized metabolites were successfully classified, according to the HMDB compounds database.

#### Analysis of MA-Induced Differential Metabolites

Variable importance plot Hierarchical clustering analysis was performed to verify the contribution degree of differential metabolites on distinguishing two groups (Figure 4F). A total of 46 differentially annotated metabolites, including 26 upregulated and 20 downregulated metabolites, exhibited significant differences in expression levels between the MA and control groups, and they all actively contributed to distinguishing the two groups (VIP > 1). Expression levels of these 46 metabolites were summarized in hierarchical cluster analysis (Figure 4G).

Kyoto Encyclopedia of Genes and Genomes analysis was performed to investigate the signaling pathways that were impacted by the differential metabolites. In total, 46 differential metabolites were mainly enriched in 20 pathways (Figure 4H). Ten of these signaling pathways were significantly altered following MA treatment. These pathways included the Sphingolipid signaling pathway, Sphingolipid metabolism, Peroxisome Proliferators-Activated Receptors (PPAR) signaling pathway, Synaptic vesicle cycle, Gap junction pathway, Insulin resistance, Valine, leucine, and isoleucine biosynthesis pathways.

### Correlative Analysis Between Gut Microbiota and Serum Metabolites

Spearman’s analysis revealed that the 46 differentially annotated metabolites were correlated with differential microbiota. The top 50 correlated differential microbiota, such as f_Bacteroidales_S24-7_group, f_Ruminococcaceae, f_Bifidobacteriaceae, f_Prevotellaceae, f__Lactobacillaceae, and f__Lachnospiraceae presented a close association with these 46 metabolites (Figure 4I). Based on the Procrustes analysis of microbial and metabolomics data, characteristics of microbial PCA were significantly consistent with metabolite expression (Figure 4J).

## Discussion

In this study, MA induced locomotor sensitization and depression/anxiety-like behaviors in mice, and stimulated striatal neurotoxicity, including dysfunction of dopaminergic system, neural apoptosis, and autophagy. Moreover, MA obviously reduced microbial diversity and altered the component of microbiota. RNA-seq results revealed that MA treatment stimulated colonic inflammation, and subsequently, the TLR4-MyD88-NF-κB pathway was confirmed to be activated, along with weakening of barrier functions. In addition, serum metabolome was altered after MA treatment. Specifically, sphingolipids and serotonin metabolism were reshaped. These findings imply that MA-induced dysbiosis of gut homeostasis might modulate neurotoxicity through the serum metabolism pathway.

### MA-Induced Dopaminergic Dysfunction, Neural Apoptosis, and Autophagy Are Closely Associated With Locomotor Sensitization and Depression/Anxiety-Like Behaviors

Elevated striatal DA and DAT is vital for MA-induced locomotor sensitization (Shin et al., 2017). Therefore, the function of striatum dopaminergic system was evaluated in this study. Accompanied by behavioral phenotypes, expression levels of DAT and TH were significantly upregulated in the striatum after MA treatment, suggesting that MA induced the hyperactivity of the dopaminergic system and triggered the release of striatal DA. Indeed, acute amphetamine exposure can activate the DA system (Wang et al., 2010). In our regimen, mice experienced 2 days of withdrawal before the last MA injections, which explains hyperactivity of the DA system. Meanwhile, MAOA, the major clastic enzyme of dopamine (Yeung et al., 2019), was significantly upregulated, suggesting accelerated dopamine loss.

Autophagy is essential in regulating dopamine release and attenuates dopamine deficits in various neurodegenerative diseases (Limanaqi et al., 2019). Our results showed stimulated neural autophagy, suggesting its regulatory effects on MA-induced dopaminergic dysfunction. Moreover, MA aroused apoptosis by elevating the expressions of p53, Bax, caspase 3, and cleaved caspase 3, which have been reported to mediate neuro-apoptosis after MA-analog methiopropamine treatment, and ultimately cause dopaminergic neurodegeneration (Nguyen et al., 2019). This phenomenon suggests close associations between the dopaminergic system and neuro-apoptosis. Furthermore, MA can promote apoptosis by stimulating the PI3K/AKT signaling pathway, which then activates the extracellular signal-regulated kinase (ERK) 1/2, an enzyme that triggers MA-induced hyperactivity and behavioral sensitization (Tsokas et al., 2007; Yan et al., 2014). Accordingly, it is believable that MA induced-neuronal autophagy and apoptosis could modulate the dopaminergic dysfunction, which further mediated locomotor sensitization.

After chronic MA incubation, sudden withdrawal can induce depression/anxiety-like behaviors (Jacobskind et al., 2019). These behavioral phenotypes were also triggered by MA in this study. Given the stimulation of neural autophagy and apoptosis, potential mechanisms of MA-induced depression/anxiety-like behaviors seemed to be explainable. In a rodent model, amyloid Beta-treated rats showed elevated autophagy and depression/anxiety-like behaviors, while alleviation of autophagy through silibinin protected against these abnormal behaviors (Song et al., 2017). Similarly, neural apoptosis promotes the development of depression/anxiety. By suppressing neuronal apoptosis, tertiary butylhydroquinone alleviates MA-induced depression-like behaviors (Meng et al., 2020). In bacteria-induced anxiety/depression models, inhibition of apoptosis was shown to effectively improve these behavioral phenotypes (Shal et al., 2019). These findings imply that neuronal autophagy and apoptosis might play crucial roles in MA-induced depression/anxiety-like behaviors.

### MA Disturbed Gut Homeostasis by Disordering Microbiome and Stimulating Colonic Inflammation

Regulatory effects of gut microbiota on the CNS have been widely reported (Chevalier et al., 2020). In this study, gut microbiome was restructured after MA treatment, including increased Bacteroidetes, decreased Firmicutes, Lachnospiraceae, and Ruminococcaceae abundances. These alterations could be associated with the dopaminergic system, which further modulates locomotor sensitization. A higher abundance of Bacteroidetes promotes dopamine production (Gong et al., 2020), while Firmicutes alter dopamine agonist quinpirole-induced locomotor sensitization (Jung et al., 2018). In attention-deficit/hyperactivity disorder, decreased Lachnospiraceae and Ruminococcaceae abundance affect neural dopamine levels through the gut-brain axis (Wan et al., 2020).

Moreover, the abundance of Lactobacillaceae and Bifidobacteriaceae after MA treatment were altered. In depressed patients, the abundance of gut bifidobacteria and lactobacilli were significantly reduced, a phenomenon that was alleviated by turnover trials (Han and Kim, 2019; Heym et al., 2019). Animal experiments have confirmed that Lactobacillus and Bifidobacterium improve immobilization stress-induced depression/anxiety and alleviate microbial dysbiosis (Han and Kim, 2019), implying that dysbiosis of Lactobacillaceae and Bifidobacteriaceae contribute to MA-induced depression/anxiety. In addition, there could be correlations between dysregulated probiotics and MA-induced neurotoxicity. Supplementations of these probiotics can suppress LPS-induced hippocampal apoptosis by inhibiting expression levels of cleaved caspase 3, while downregulating Bax and Bax/Bcl-2 ratios (Mohammadi et al., 2019). In accordance with our previous study (Chen et al., 2021), MA upregulated Bifidobacterium and downregulated Lactobacillus. We hypothesized that it might exist the functional differences between these two probiotics and the compensatory increase in Bifidobacterium could be a feedback protective mechanism.

Microbial dysbiosis could play crucial roles in MA-induced colonic inflammation. KEGG analysis revealed that inflammation-related pathways were mostly enriched, while second level term of gene ontology revealed that bacterial infectious diseases were enriched. In addition, the TLR4-MyD88-NF-κB pathway, which is a specific target of Gram-negative bacterial product-LPS (Płóciennikowska et al., 2015), was stimulated after MA treatment. Furthermore, the decreased Ruminococcaceae and Lachnospiraceae abundance in our study has also been observed in IBD patients (Humbel et al., 2019), implying its strong association with disease development. The IBD pathway was also enriched in the RNA-sequencing results. In fact, as the dysfunction of intestinal barrier, normal microbiota can also induce intestinal inflammation, which is attenuated by restoration of intestinal barrier (Guo et al., 2019; Chen et al., 2020). These findings indicate microbial involvement in MA-induced intestinal inflammation.

In a state of intestinal inflammation, immune responses produce various transmitters, including proinflammatory cytokines and metabolites (Dantzer, 2006). Along with intestinal inflammation, its barrier functions can be impaired, which allows these products and microbial metabolites into systemic blood circulation (Hsiao et al., 2013). Through the blood–brain barrier, these molecules can send signals to the brain, resulting in serious behavioral changes (Waclawiková and El, 2018). Correspondingly, MA induced serious colonic inflammation and barrier impairment, which might partly impact on locomotor sensitization and depression/anxiety-like behaviors through serum metabolism. In fact, patients with IBD usually present concomitant symptoms of depression/anxiety. Alleviation of intestinal inflammation through probiotics supplementation improves these abnormal behaviors (Emge et al., 2016; Abautret-Daly et al., 2018). These evidence suggest that improvement of intestinal inflammation could be a potential strategy for managing MA-induced CNS disorders.

### MA Treatment Reshaped Serum Metabolome, Characterized by Sphingolipids and Serotonin Alterations

Bacteroides-derived sphingolipids are vital in maintaining intestinal homeostasis and symbiosis (Brown et al., 2019). Here, sphingolipids were mostly altered after MA treatment. The sphingolipid metabolism pathway was also enriched in KEGG analysis of serum metabolites, as previously reported. In fact, sphingolipid-mediated metabolic and immune signaling events have been implicated in autoimmune and chronic inflammatory diseases, including IBD (Nixon, 2009; Brown et al., 2019). These findings strongly imply the involvement of sphingolipids in MA-induced intestinal inflammation. In addition to regulation of intestinal inflammation, sphingolipids have been closely associated with depression and anxiety. Depression and anxiety are correlated with higher activities of sphingolipid metabolizing enzymes, which catalyze the hydrolysis of sphingomyelin to ceramide and phosphorylcholine (Zoicas et al., 2020). In this study, levels of major serum sphingolipids were suppressed by MA, suggesting its potential mechanism in depression/anxiety in mice. Furthermore, sphingolipids have emerged the manipulation to dopamine D1 receptor while dopamine activity can be regulated by sphingomyelinase (Mystek et al., 2016). This explains the mechanisms through which MA-induces dysfunctions of the dopaminergic system, although detailed mechanisms have not been established. Sphingolipids have also been shown to regulate cellular apoptosis and macroautophagy (Piccinini et al., 2010; Pujol-Lereis, 2019), which were initiated by MA in this study. These findings indicate that sphingolipid metabolism is a potential microbial mechanism for MA-induced neurotoxicity.

Enterogenous serotonin is the largest source of serotonin to the host and is recognized as the crucial medium of gut-brain axis (Gheorghe et al., 2019; Martin et al., 2019). In fact, gut microbiota can regulate host immunity by modulating serotonin metabolism. IBD patients have low serum serotonin levels, and its supplementation prevents or alleviates intestinal inflammation (Gao et al., 2018; Stasi et al., 2019). In this study, serum serotonin was reduced after MA treatment, implying its close association with MA-induced intestinal inflammation. Furthermore, MA can result in serotonin depletion and further influence locomotor sensitization (Huang et al., 2019), which were in accordance with our results. Knockout of serotonin transporter inhibits MA-induced locomotor sensitization, which is restored by selective 5-HT1B antagonist receptor (Igari et al., 2015). Meanwhile, suppressed serotonin level is closely associated with depression/anxiety and selective serotonin reuptake inhibitors have been widely used in depression/anxiety treatment (DeFilippis and Wagner, 2014; Yuan et al., 2015). Given the observed depression/anxiety-like behaviors, we postulated that enterogenous serotonin modulated MA-induced behavioral alterations through serum metabolism. In addition, serotonin reuptake disorders in depression can cause astrocytic apoptosis (Bansal et al., 2019). Moreover, autophagic dysfunction and apoptosis activation in traumatic brain injury can be reversed by N-acetyl serotonin (Rui et al., 2020), suggesting that decreased serotonin level could be involved in MA-induced neural apoptosis and autophagy. Finally, serotonin has been confirmed to control DA release (Boureau and Dayan, 2011), suggesting that a decrease in serotonin level could be the cause of MA-induced dopaminergic dysfunction.

## Limitations and Conclusion

It is necessary to note some limitations of this study. First, our microbial results revealed reverse alterations in the abundance of probiotics Lactobacillaceae and Bifidobacteriaceae, suggesting slight differences in their biologic functions. The available evidence cannot explain this phenomenon. Second, the fecal microbiota transplantation experiment is the effective method for verifying the regulatory effects of microbiota, which we plan to perform in a future study. However, the detection of serum metabolism revealed altered microbial metabolites, which partly make up for the deficiency of our study design. Another limitation is that although studies have reported on the effects of regulation of serum metabolism on brain behaviors, metabolic mechanisms, and signaling pathways involved remain to be identified. Finally, our experiment was conducted on the animal model, and this limited the accurately mirror of human data. Further studies are needed to evaluate the fecal and serum samples from MA abusers.

**Figure 5:**
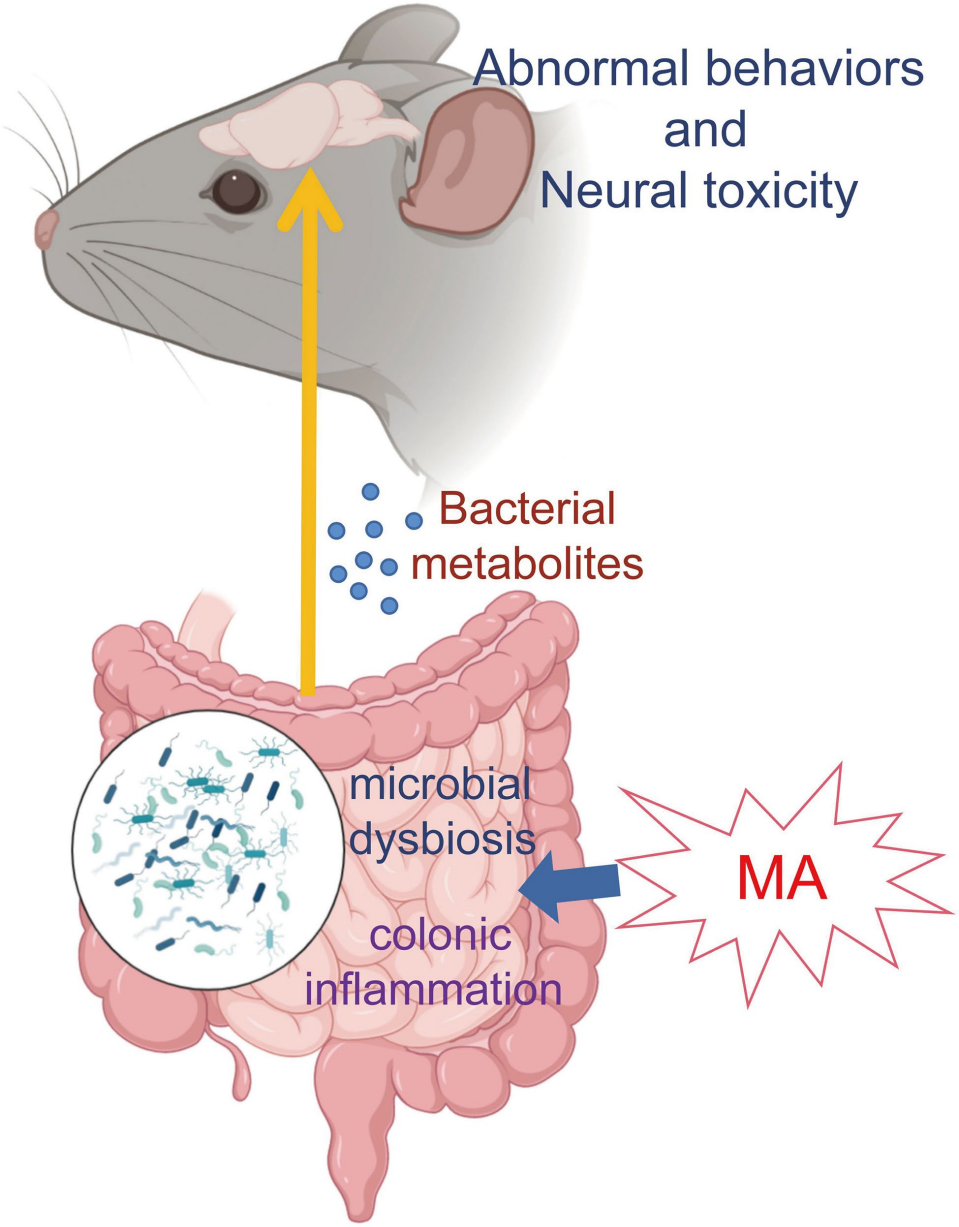
Mechanism map. MA disordered the gut homeostasis *via* disturbing microbiome and stimulating inflammation. Then, microbial metabolites entered the systemic circulation through the damaged intestinal barrier, which was involved in MA-induced neurotoxicity and behavioral alterations.

These findings indicate that MA could promote intestinal inflammation and damage its barrier functions, which allows metabolites from dysbiosis microbiota into systemic circulation. In addition, microbial metabolites (especially sphingolipids and serotonin) could be a potential pathway to participate in MA-induced neurotoxicity, although the detailed mechanisms need to be further investigated (Figure 5). In summary, this study provides metabolic clues regarding the interactions between gut homeostasis and the CNS, which contribute to a better understanding of MA-induced neurotoxicity.

## Data Availability Statement

The datasets presented in this study can be found in online repositories. The name of the repository and accession numbers can be found at: SRA, NCBI; PRJNA753280 and PRJNA813296.

## Ethics Statement

The animal study was reviewed and approved by National Institute of Health Guide for the Care and Use of Laboratory Animals of the Southern Medical University. The number of the Ethical Committee Approval Code was L2018123.

## Author Contributions

X-LX and QW contributed to conception and design of the study. K-KZ and L-JC organized the database. L-BW and X-WL performed the statistical analysis. K-KZ and J-LL wrote the first draft of the manuscript. J-HL, L-LX, and J-ZY wrote sections of the manuscript. All authors contributed to the article and approved the submitted version.

## Funding

This work was supported by the Guangdong Natural Science Foundation under grant no. 2020A1515010370 and 2021A1515012456; Department of Science and Technology of Guangzhou city under grant no. 202002030043.

## Conflict of Interest

The authors declare that the research was conducted in the absence of any commercial or financial relationships that could be construed as a potential conflict of interest.

## Publisher’s Note

All claims expressed in this article are solely those of the authors and do not necessarily represent those of their affiliated organizations, or those of the publisher, the editors and the reviewers. Any product that may be evaluated in this article, or claim that may be made by its manufacturer, is not guaranteed or endorsed by the publisher.

## Footnotes

^1^ http://drive5.com/uparse/

^2^ http://rdp.cme.msu.edu/

^3^ https://github.com/jstjohn/SeqPrep

^4^ https://github.com/najoshi/sickle

^5^ http://tophat.cbcb.umd.edu/

^6^ http://www.geneontology.org/

^7^ https://www.genome.jp/kegg/

^8^ https://string-db.org/

^9^ http://david.ncifcrf.gov

^10^ http://www.hmdb.ca/

^11^ https://metlin.scripps.edu/
