# Supplementary material for: Methamphetamine Disturbs Gut Homeostasis and Reshapes Serum Metabolome, Inducing Neurotoxicity and Abnormal Behaviors in Mice
Source: Front Microbiol. 2022 Apr 18;13:755189. doi: 10.3389/fmicb.2022.755189 (PMC9058162; doi:10.3389/fmicb.2022.755189)
Supplement: Supplementary file 1 [file Table_2.DOCX]

**Supplementary Table 1**

| **Pathway Enrichment** | | | | | | |
| --- | --- | --- | --- | --- | --- | --- |
| **NO** | **Pathway ID** | **Pathway** | **COUNT** | **%** | **%** | **Pvalue** |
| 1 | mmu05168 | Herpes simplex infection | 22 | H2-T24, SP100, H2-EB1, IL15, STAT1, DDX58, STAT2, H2-Q6, H2-Q7, OAS1A, TAP1, IFIT1BL1, IFIT1, H2-Q1, OAS1G, C3, SOCS3, PER3, CCL5, IRF9, H2-D1, TLR2 | 5.583756345 | 3.53E-10 |
| 2 | mmu04145 | Phagosome | 14 | H2-T24, H2-EB1, C1RA, H2-Q6, H2-Q7, TAP1, THBS2, H2-Q1, C3, FCGR4, MRC1, CD14, H2-D1, TLR2 | 3.553299492 | 2.31E-05 |
| 3 | mmu05150 | Staphylococcus aureus infection | 8 | C3, C4B, H2-EB1, C1RA, FCGR4, CFB, ICAM1, C2 | 2.030456853 | 4.23E-05 |
| 4 | mmu05164 | Influenza A | 12 | IL33, CXCL10, SOCS3, H2-EB1, DDX58, STAT1, CCL5, STAT2, OAS1A, IRF9, ICAM1, OAS1G | 3.045685279 | 4.36E-04 |
| 5 | mmu05416 | Viral myocarditis | 8 | H2-T24, H2-EB1, CAV1, H2-Q6, H2-Q7, H2-Q1, H2-D1, ICAM1 | 2.030456853 | 7.72E-04 |
| 6 | mmu04623 | Cytosolic DNA-sensing pathway | 7 | ZBP1, IL33, CXCL10, RIPK3, DDX58, CCL5, TREX1 | 1.776649746 | 0.001372629 |
| 7 | mmu05332 | Graft-versus-host disease | 6 | H2-T24, H2-EB1, H2-Q6, H2-Q7, H2-Q1, H2-D1 | 1.52284264 | 0.003096384 |
| 8 | mmu04610 | Complement and coagulation cascades | 7 | C3, C4B, C1RA, PLAT, TFPI, CFB, C2 | 1.776649746 | 0.003315268 |
| 9 | mmu05330 | Allograft rejection | 6 | H2-T24, H2-EB1, H2-Q6, H2-Q7, H2-Q1, H2-D1 | 1.52284264 | 0.00428013 |
| 10 | mmu05160 | Hepatitis C | 9 | SOCS3, DDX58, STAT1, STAT2, OAS1A, IFIT1, IFIT1BL1, IRF9, OAS1G | 2.284263959 | 0.004603139 |
| 11 | mmu04612 | Antigen processing and presentation | 7 | H2-T24, H2-EB1, H2-Q6, H2-Q7, TAP1, H2-Q1, H2-D1 | 1.776649746 | 0.004834058 |
| 12 | mmu04940 | Type I diabetes mellitus | 6 | H2-T24, H2-EB1, H2-Q6, H2-Q7, H2-Q1, H2-D1 | 1.52284264 | 0.006614743 |
| 13 | mmu05140 | Leishmaniasis | 6 | C3, TGFB2, H2-EB1, FCGR4, STAT1, TLR2 | 1.52284264 | 0.007559382 |
| 14 | mmu05320 | Autoimmune thyroid disease | 6 | H2-T24, H2-EB1, H2-Q6, H2-Q7, H2-Q1, H2-D1 | 1.52284264 | 0.01160228 |
| 15 | mmu04514 | Cell adhesion molecules (CAMs) | 9 | H2-T24, H2-EB1, H2-Q6, H2-Q7, MADCAM1, H2-Q1, H2-D1, ITGA9, ICAM1 | 2.284263959 | 0.012736025 |
| 16 | mmu05144 | Malaria | 5 | TGFB2, ACKR1, THBS2, ICAM1, TLR2 | 1.269035533 | 0.013297377 |
| 17 | mmu05410 | Hypertrophic cardiomyopathy (HCM) | 6 | TGFB2, DES, TNNT2, TPM2, TPM1, ITGA9 | 1.52284264 | 0.017785679 |
| 18 | mmu04510 | Focal adhesion | 10 | COL1A1, COL3A1, TNXB, CAV1, PDGFD, THBS2, MYL9, ARHGAP35, ITGA9, MYLK | 2.538071066 | 0.01822172 |
| 19 | mmu04668 | TNF signaling pathway | 7 | CXCL10, SOCS3, RIPK3, IL15, CCL5, IFI47, ICAM1 | 1.776649746 | 0.018363754 |
| 20 | mmu05152 | Tuberculosis | 9 | C3, TGFB2, H2-EB1, FCER1G, FCGR4, STAT1, MRC1, CD14, TLR2 | 2.284263959 | 0.020058805 |
| 21 | mmu05323 | Rheumatoid arthritis | 6 | TGFB2, H2-EB1, IL15, CCL5, ICAM1, TLR2 | 1.52284264 | 0.020575841 |
| 22 | mmu05414 | Dilated cardiomyopathy | 6 | TGFB2, DES, TNNT2, TPM2, TPM1, ITGA9 | 1.52284264 | 0.021565966 |
| 23 | mmu05134 | Legionellosis | 5 | C3, NAIP5, NAIP6, CD14, TLR2 | 1.269035533 | 0.023655034 |
| 24 | mmu05321 | Inflammatory bowel disease (IBD) | 5 | TGFB2, H2-EB1, STAT1, RORC, TLR2 | 1.269035533 | 0.026469729 |
| 25 | mmu04975 | Fat digestion and absorption | 4 | ABCG5, PLA2G2A, PLA2G3, APOB | 1.015228426 | 0.036061629 |
| 26 | mmu04622 | RIG-I-like receptor signaling pathway | 5 | CXCL10, RNF125, DDX58, DHX58, ISG15 | 1.269035533 | 0.041545779 |
| 27 | mmu05169 | Epstein-Barr virus infection | 7 | H2-T24, DDX58, H2-Q6, H2-Q7, H2-Q1, H2-D1, ICAM1 | 1.776649746 | 0.047012451 |
| 28 | mmu05162 | Measles | 7 | DDX58, STAT1, STAT2, OAS1A, IRF9, OAS1G, TLR2 | 1.776649746 | 0.047012451 |
| 29 | mmu04976 | Bile secretion | 5 | ABCG5, AQP4, SLC51B, ABCB1A, AQP1 | 1.269035533 | 0.047457813 |

KEGG analysis was performed on the DEGs. 414 MA-induced DEGs were mainly enriched into 29 pathways (P-value < 0.05). Also, the number and the ratio of DEGs in every pathways have been shown.
