# Supplementary material for: Methamphetamine Disturbs Gut Homeostasis and Reshapes Serum Metabolome, Inducing Neurotoxicity and Abnormal Behaviors in Mice
Source: Front Microbiol. 2022 Apr 18;13:755189. doi: 10.3389/fmicb.2022.755189 (PMC9058162; doi:10.3389/fmicb.2022.755189)
Supplement: Supplementary file 2 [file Table_3.DOCX]

**Supplementary Figure. 1**


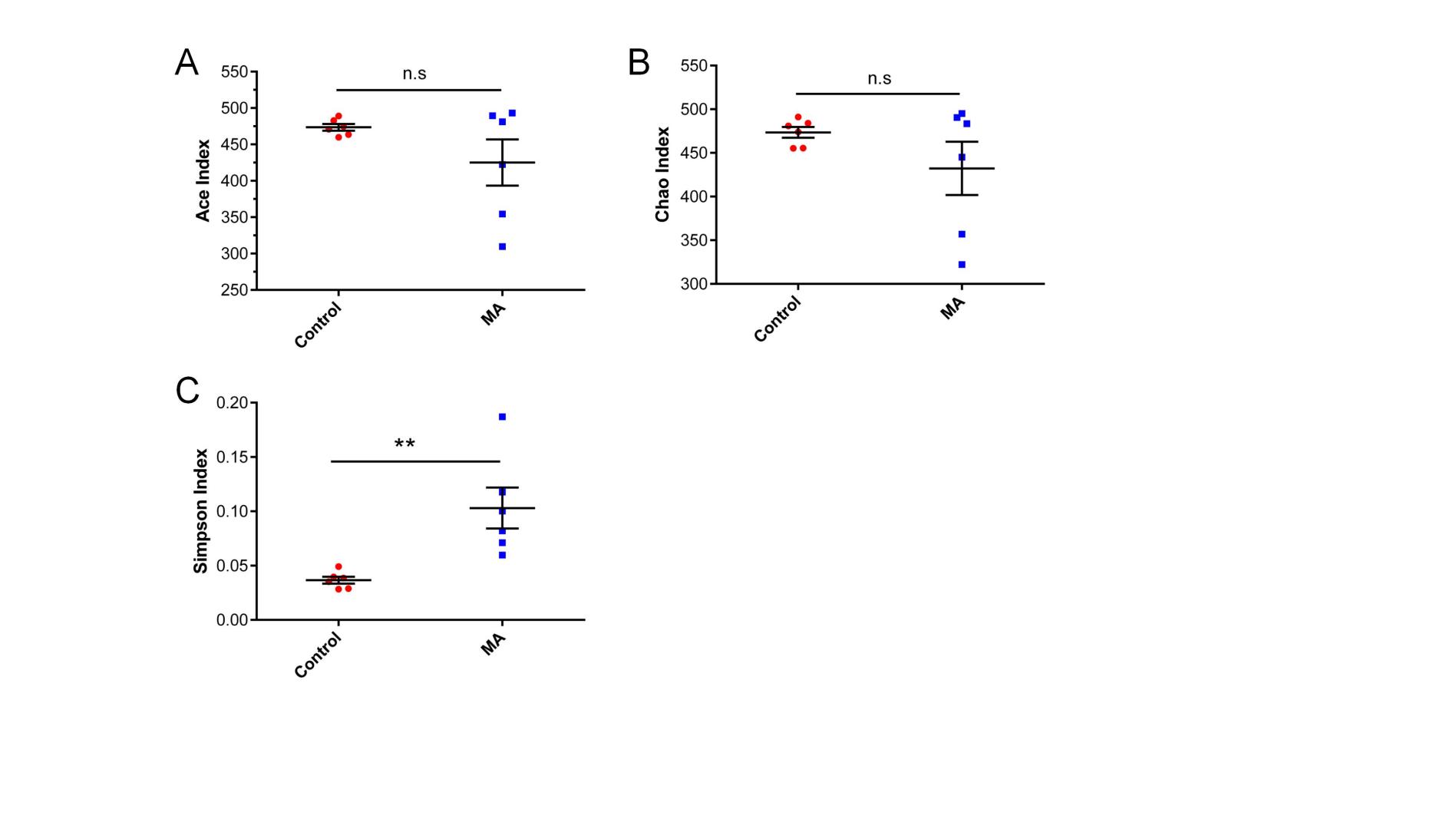


Assessment of the difference in richness and diversity between the MA group and the control group. Microbial richness as evaluated by Ace index and Chao index (A, B), microbial diversity as evaluated by Simpson index (C). Increase in the Simpson index indicates the reduction of microbial diversity (**P < 0.01).
